# Brachiopoda of Greece: an annotated checklist

**DOI:** 10.3897/BDJ.4.e8169

**Published:** 2016-11-01

**Authors:** Vasilis Gerovasileiou, Nicolas Bailly

**Affiliations:** ‡ Institute of Marine Biology, Biotechnology and Aquaculture, Hellenic Centre for Marine Research, Heraklion, Greece

**Keywords:** Aegean Sea, Sea of Crete, Levantine Sea, Ionian Sea, Eastern Mediterranean

## Abstract

**Background:**

Until today, only scattered species records of Brachiopoda from Greece have been included in publications on the Mediterranean brachiopod fauna. These records were mostly based on material collected during marine expeditions in the eastern Mediterranean decades ago, while few recent additional records appear in ecological studies. The aim of this paper was to give the first checklist of brachiopod species of Greece, in the framework of the Greek Taxon Information System (GTIS) initiative of the LifeWatchGreece Research Infrastructure (ESFRI), by reviewing the existing literature.

**New information:**

Twelve brachiopod species have been found in Greek waters so far. The nomenclature, distribution, fossil records, ecology, and literature sources are discussed for each species.

## Introduction

Until Logan’s revision of the Mediterranean Brachiopoda (Logan 1979), several records of their extant taxa from the eastern Mediterranean were scattered in old publications on molluscs and in general benthic studies (see Logan et al. 2002 for a detailed publication list). Brachiopod records from Greece were mostly based on material that was collected during the oceanographic expeditions of the French research vessels "Calypso" and "Jean Charcot" in the 1950’s and 1960’s (Logan 1979, Logan et al. 2002). Since then, few ecological studies on the sciaphilic hard substrate communities reported on Brachiopoda from Greek waters (e.g. Antoniadou and Chintiroglou 2005, Taviani et al. 2011, Gerovasileiou et al. 2015). The aim of the present study was to give an updated, annotated list of Brachiopoda of the Greek seas.

## Materials and methods

The checklist of Brachiopoda of Greece (Suppl. material 1) was created in the framework of the Greek Taxon Information System (GTIS) initiative of the LifeWatchGreece Research e-Infrastructure (Bailly et al. 2016, this special collection). Its construction was initially based on the species lists of Mediterranean Brachiopoda compiled by Logan (1979) and Logan et al. (2002). All records in these lists were cross-checked against the World Register of Marine Species (WoRMS Editorial Board 2016) and the Brachiopoda Database (Emig et al. 2016) in order to update taxonomy and nomenclature. Records and information published since 2002 were incorporated in the list. The list is annotated with information regarding species distribution in the Greek seas (i.e. North Aegean, South Aegean, Levantine Sea, Ionian Sea), ecological remarks (i.e. depth and habitat), and the relevant literature sources. Information about fossil records of Brachiopoda from Greece was extracted from Logan et al. (2004), Nielsen et al. (2006), Koskeridou (2007), and references therein. The classification follows WoRMS and Emig et al. (2013).

## Checklists

### List of brachiopod species found in Greek waters

#### Craniata

#### Craniida

#### Craniidae

#### Novocrania anomala

(Müller, 1776)

##### Distribution

North Aegean, South Aegean, Levantine Sea, Ionian Sea

##### Horizon

Pleistocene, Pliocene, Holocene

##### Notes

Recorded by Logan (1979), Morri et al. (1999), Logan et al. (2002), Gerovasileiou et al. (2015). Habitat/Substrate: Caves, precoralligenous, coralligenous, bioconcretions, banks, boulders, muddy sand, coarse sand. Depth: 2-150 m.

#### Novocrania turbinata

(Poli, 1795)

Novocrania turbinata Doubtful species, may be a synonym of *N. turbinata*. See discussion.

##### Distribution

South Aegean

##### Horizon

Holocene

##### Notes

Recorded by Logan and Long (2001). Habitat/Substrate: boulders. Depth: 65-150 m.

#### Rhynchonellata

#### Terebratulida

#### Cancellothyrididae

#### Terebratulina retusa

(Linnaeus, 1758)

##### Distribution

Levantine Sea

##### Horizon

Pleistocene, Pliocene, Holocene

##### Notes

Recorded by Logan (1979), Logan et al. (2002). Habitat/Substrate: Grey-yellow muds. Depth: 421 m. Remarks: Species record was based on a single poorly preserved specimen.

#### Kraussinidae

#### Megerlia truncata

(Linnaeus, 1767)

##### Distribution

North Aegean, South Aegean, Levantine Sea, Ionian Sea

##### Horizon

Pleistocene, Pliocene, Miocene, Holocene

##### Notes

Recorded by Logan (1979), Logan et al. (2002), Taviani et al. (2011). Habitat/Substrate: Rocks, precoralligenous, coralligenous, bioconcretions, *Madrepora*-*Lophelia* rudstone, gravel, shells, detritus, coarse sand, muddy sand, mud. Depth: 29-762 m.

#### Megathyrididae

#### Argyrotheca cistellula

(Wood, 1841)

##### Distribution

South Aegean, Levantine Sea, Ionian Sea

##### Horizon

Pleistocene, Holocene

##### Notes

Recorded by Logan (1979), Logan et al. (2002). Habitat/Substrate: Caves and overhangs. Depth: 6-30 m.

#### Argyrotheca cuneata

(Risso, 1826)

##### Distribution

North Aegean, South Aegean, Levantine Sea, Ionian Sea

##### Horizon

Pleistocene, Holocene

##### Notes

Recorded by Logan (1979), Simboura et al. (1995), Morri et al. (1999), Logan et al. (2002). Habitat/Substrate: Caves, overhangs, offshore rocks, coralligenous, bioconcretions, banks, gravel, coarse sand, muddy sand. Depth: 2-210 m.

#### Joania cordata

(Risso, 1826)

##### Distribution

North Aegean, South Aegean, Levantine Sea, Ionian Sea

##### Horizon

Pleistocene, Pliocene, Holocene

##### Notes

Recorded by Logan (1979), Simboura et al. (1995), Morri et al. (1999), Logan et al. (2002). Habitat/Substrate: Caves, overhangs, offshore rocks, coralligenous, bioconcretions, banks, gravel, coarse sand. Depth: 6-135 m.

#### Megathiris detruncata

(Gmelin, 1791)

##### Distribution

North Aegean, South Aegean, Levantine Sea, Ionian Sea

##### Horizon

Pleistocene, Pliocene, Holocene

##### Notes

Recorded by Logan (1979), Morri et al. (1999), Logan et al. (2002), Antoniadou and Chintiroglou (2005). Habitat/Substrate: Caves, overhangs, rocky shoals, precoralligenous, coralligenous, bioconcretions, banks, gravel, mud. Depth: 10-180 m.

#### Platidiidae

#### Platidia anomioides

(Scacchi & Philippi, 1844, in Philippi, 1844)

##### Distribution

Levantine Sea

##### Horizon

Holocene

##### Notes

Recorded by Logan (1979), Logan et al. (2002). Habitat/Substrate: mud. Depth: 115-180 m.

#### Platidia davidsoni

(Eudes-Deslongchamp, 1885)

Platidia davidsoni Doubtful species, may be a synonym of *P. anomioides*. See discussion.

##### Distribution

North Aegean

##### Horizon

Holocene

##### Notes

Recorded by Logan (1979). Habitat/Substrate: Coralligenous. Depth: 108-112 m.

#### Terebratulidae

#### Gryphus vitreus

(Born, 1778)

##### Distribution

North Aegean, South Aegean, Levantine Sea, Ionian Sea

##### Horizon

Holocene

##### Notes

Recorded by Logan (1979), Logan et al. (2002), Taviani et al. (2011). Habitat/Substrate: *Madrepora*-*Lophelia* rudstone, *Lophelia*-*Madrepora* rubble, pelagic mudstone and wackestone, silty sand, sandy mud, mud. Depth: 130-2,133 m.

#### Incertae familiae

#### Gwynia capsula

(Jeffreys, 1859)

##### Distribution

North Aegean

##### Horizon

Holocene

##### Notes

Recorded by Antoniadou and Chintiroglou (2005). Habitat/Substrate: Hard substrate in sciaphilic algal community. Depth: 15-40 m.

## Discussion

The checklist of Brachiopoda of Greece comprises 12 species classified into 9 genera, 6 families, 2 orders, and 2 classes. *Novocrania turbinata* and *Platidia davidsoni* have been included in the list as doubtful species. Logan and Long (2001) separated the species *N. turbinata* and *N. anomala*, which had been synonymized for a long period. Emig (2014) made a synthesis on the issue and, on the basis of molecular data analyzed by Cohen et al. (2014), he concluded that the two species were synonyms. However, he mentions in his discussion that the lack of comprehensive study on morphological characters hinders the conclusion, especially concerning the eastern Mediterranean basin. The synonymy of *P. davidsoni* with *P. anomioides* has been also questioned (A. Logan, pers. comm.). Thus, we chose to keep the two species in the list until their status is completely clarified.

The only recent addition to the brachiopod diversity of Greece is the species *Gwynia capsula* (Antoniadou and Chintiroglou 2005). Logan et al. (2004) had attributed the fact that this species was not found in the eastern basin either to the eastward Mediterranean faunal impoverishment or to the limited research effort in this area on this phylum. Indeed, *G. capsula* was reported one year later by Antoniadou and Chintiroglou (2005), on hard substratum sciaphilic assemblages (15-40 m) in the North Aegean. 

In the Greek seas, Brachiopoda have been recorded in habitats ranging from dimly lit hard substrata of the sublittoral zone (e.g. caves and coralligenous assemblages) to bathyal muddy bottoms and deep-water coral facies.

Overall, the brachiopod fauna of the Greek seas comprises 85% of the Mediterranean brachiopod species, being considerably richer than in the other countries of the eastern basin: 5 species have been reported to date from Turkey (Çinar 2014, Gönülal and Güreşen 2014), Cyprus, and Lebanon, and 2 species from Israel (Logan 1979, Logan et al. 2002). No endemic bachiopods have been found in the Greek seas. Nevertheless, given the low number of studies focusing on dark habitats in the area, further research is expected to increase our knowledge on the brachiopod fauna of the eastern Mediterranean.

## Supplementary Material

Supplementary material 1 Checklist of Brachiopoda of Greece Data type: Taxonomic checklist
Brief description: Taxonomic checklist of Brachiopoda known to occur in Greek waters.
File: oo_97945.xls Vasilis Gerovasileiou, Nicolas Bailly

XML Treatment for Craniata

XML Treatment for Craniida

XML Treatment for Craniidae

XML Treatment for Novocrania anomala

XML Treatment for Novocrania turbinata

XML Treatment for Rhynchonellata

XML Treatment for Terebratulida

XML Treatment for Cancellothyrididae

XML Treatment for Terebratulina retusa

XML Treatment for Kraussinidae

XML Treatment for Megerlia truncata

XML Treatment for Megathyrididae

XML Treatment for Argyrotheca cistellula

XML Treatment for Argyrotheca cuneata

XML Treatment for Joania cordata

XML Treatment for Megathiris detruncata

XML Treatment for Platidiidae

XML Treatment for Platidia anomioides

XML Treatment for Platidia davidsoni

XML Treatment for Terebratulidae

XML Treatment for Gryphus vitreus

XML Treatment for Incertae familiae

XML Treatment for Gwynia capsula
